# Supplementary material for: Mesoporous Silica Particle as an RNA Adsorbent for Facile Purification of In Vitro-Transcribed RNA
Source: Int J Mol Sci. 2023 Aug 3;24(15):12408. doi: 10.3390/ijms241512408 (PMC10419103; doi:10.3390/ijms241512408)
Supplement: Supplementary file 1 [file ijms-24-12408-s001.zip › ijms-2545477-supplementary.pdf]

Supplementary Materials

Mesoporous Silica Particle as RNA Adsorbent for Facile Purification of In Vitro-Transcribed RNA

Eunbin Cho, Jayoung Namgung, Jong Sam Lee, Jinmin Jang, Bong-Hyun Jun and Dong-Eun Kim *

Department of Bioscience and Biotechnology, Konkuk University, 120 Neungdong-ro, Gwangjin-gu, Seoul 05902, Republic of Korea
* Correspondence: kimde@konkuk.ac.kr.

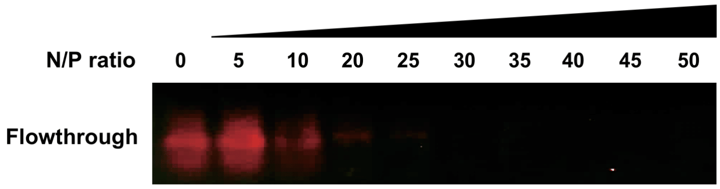

Figure S1. Adsorption of IVT RNA present in the post-*in vitro* transcription reaction mixture to mesoporous silica in the presence of spermidine. The unpurified IVT RNA (10 μg) supplemented with spermidine at increasing N/P ratio was purified by spin column filled with mesoporous silica using elution buffer containing 10 mM EDTA. Spermidine at different N/P ratios were tested for binding of the unpurified IVT RNA after in vitro transcription reaction. Aliquots of the flowthrough were analyzed on 1.0% agarose gel pre-stained with GelRed, and the RNA present in each fraction was visualized under UV illumination.
